# Identification of Rice Blast Loss-of-Function Mutant Alleles in the Wheat Genome as a New Strategy for Wheat Blast Resistance Breeding

**DOI:** 10.3389/fgene.2021.623419

**Published:** 2021-05-19

**Authors:** Huijun Guo, Qidi Du, Yongdun Xie, Hongchun Xiong, Linshu Zhao, Jiayu Gu, Shirong Zhao, Xiyun Song, Tofazzal Islam, Luxiang Liu

**Affiliations:** ^1^National Engineering Laboratory for Crop Molecular Breeding, National Center of Space Mutagenesis for Crop Improvement, Institute of Crop Sciences, Chinese Academy of Agricultural Sciences, Beijing, China; ^2^College of Life Sciences, Qingdao Agricultural University, Qingdao, China; ^3^Institute of Biotechnology and Genetic Engineering (IBGE), Bangabandhu Sheikh Mujibur Rahman Agricultural University, Gazipur, Bangladesh

**Keywords:** wheat, rice blast, wheat blast, TILLING, mutant allele, deleterious effect

## Abstract

Blast is caused by the host-specific lineages of the fungus *Magnaporthe oryzae* and is the most important destructive disease in major crop plants, including rice and wheat. The first wheat blast outbreak that occurred in Bangladesh in 2016 and the recent epidemic in Zambia were caused by the *M. oryzae Triticum* (*MoT*) pathotype, a fungal lineage belonging to *M. oryzae*. Although a few reported wheat cultivars show modest resistance to *MoT*, the patterns of genetic variation and diversity of this pathotype make it crucial to identify additional lines of resistant wheat germplasm. Nearly 40 rice blast resistant and susceptible genes have so far been cloned. Here, we used BLAST analysis to locate two rice blast susceptible genes in the wheat reference genome, *bsr-d1* and *bsr-k1*, and identified six identical homologous genes located on subgenomes A, B, and D. We uncovered a total of 171 single nucleotide polymorphisms (SNPs) in an ethyl methanesulfonate (EMS)-induced population, with mutation densities ranging from 1/1107.1 to 1/230.7 kb through Targeting Induced Local Lesions IN Genomes (TILLING) by sequencing. These included 81 SNPs located in exonic and promoter regions, and 13 coding alleles that are predicted to have severe effects on protein function, including two pre-mature mutants that might affect wheat blast resistance. The loss-of-function alleles identified in this study provide insights into new wheat blast resistant lines, which represent a valuable breeding resource.

## Introduction

Wheat blast is now a serious threat to food and nutritional security in three different continents, namely South America, Asia, and Africa (Islam et al., 2020). The first ever reported wheat blast epidemic occurred in Brazil in 1985 (Igarashi et al., 1986) and have taken place in the other South American countries in following decades, and subsequently spread to the neighboring wheat growing areas in Argentina, Bolivia, and Paraguay. In February 2016, a major outbreak affected 16% of the wheat planting area in Bangladesh, leading to an almost complete crop failure across 15000 hectares (Islam et al., 2016). Finally, during the 2017–2018 growing season, a widespread epidemic significantly affected most cultivars in both experimental and farming fields in Zambia (Tembo et al., 2020). It has been demonstrated that the pathogen *Magnaporthe oryzae pathotype Triticum* (*MoT*) was responsible for the outbreaks in both Bangladesh and Zambia, and that this lineage is closely related to those responsible for the wheat blast outbreak that occurred in South America (Islam et al., 2016; Tembo et al., 2020). Ceresini et al. (2018) assumed that wheat blast disease was introduced in Bangladesh through wheat grain trading from Brazil. In fact, previous research has shown that *M. oryzae* jumped from a native grass host to wheat during the 1980s in Brazil, after which a mutation in one of the isolates causing increased pathogenicity and the functional loss of resistance genes led to widespread *MoT* in wheat cultivars (Inoue et al., 2017).

Due to the relatively recent emergence of *Triticum*, there are only a few known resistant (R) genes available against this destructive pathogen in natural wheat varieties or germplasm (Islam et al., 2020). Beyond the well-characterized 2NS/2AS translocation genotypes that were acquired from *Aegilops ventricosa* (Cruz et al., 2016b; Cruppe et al., 2019; Juliana et al., 2020), the genes Rmg8 and RmgGR119, from the Albanian accession GR119, seemingly confer high blast resistance at both the heading stage and under high temperature conditions (Anh et al., 2015; Wang et al., 2018). While these genes are crucial to the current efforts to breed blast resistant wheat varieties, it has been shown that Rmg8 can be suppressed by *MoT*’s effector gene PWT4 (Inoue et al., 2020), and that resistance of 2NS translocation was eroded by new *MoT* virulence groups (Cruz et al., 2016a), which means other resistant mechanisms might become obsolete with the evolution of *MoT* in the near future. Hence, it is urgent to develop durable blast resistant wheat varieties and, especially, to identify novel non-2NS R genes in order to effectively control the threat posed by *MoT*. One possibility is through mutation induction, a mechanism that has been shown to be effective in creating novel alleles (Campbell et al., 2012; Lu et al., 2018) and germplasms (Xiong et al., 2017; Guo et al., 2019), and that can also be used to generate new *MoT* resistant varieties.

Over one hundred rice blast R and susceptible (S) genes and QTLs have so far been discovered or cloned, including *Ptr*, *Pi-ta*, *Pi-b*, and *Pi-21* (Srivastava et al., 2017; Zhao et al., 2018). In the case of the S gene *Pi-21* (Os04g0401000), the simultaneous deletions of 18- and 48-bp confer non-specific and durable resistance to rice blast. However, the gene is tightly linked with a locus associated with poor eating quality, which makes its use less than ideal to improve disease resistance (Fukuoka et al., 2009). Another example is BSR-K1, a protein that contains five tetratricopeptide repeats (TPRs) and binds to the mRNA of defense-related genes. The genotypes that encode for *Bsr-k1* (*Os10g0548200*) are susceptible to rice blast, while those encoding the *bsr-k1* allele, a pre-mature termination mutation, show broad resistance against both blast and bacterial blight (Zhou et al., 2018). Finally, *bsr-d1* (*Os03g32230*) is a loss of function allele that confers broad spectrum rice blast resistance in natural rice varieties. The gene encodes a putative C2H2-like transcription factor in the nucleus and is regulated by a MYB family transcription factor. Importantly, in this case, no unfavorable genes are known to be closely linked (Li W. et al., 2017).

Loss-of-function mutations therefore represent one of the ways to obtain fungal disease resistance in both natural populations and breeding scenarios. One example is the well-known *Fhb1* (*His*), a gene which encodes a histidine-rich calcium-binding protein and that originated in the lower reaches of the Yangtze Valley of China. The gene contains a 752-bp deletion within its 5′ end that confers resistance against Fusarium head blight (Li et al., 2019) and has been utilized worldwide as one of the best genetic resources in wheat breeding (Hao et al., 2020). Another example is the mildew resistant locus o (*mlo*) where resistance-conferring missense and knockout mutations against powdery mildew were induced in the conserved region of the gene by ethyl methanesulfonate (EMS) mutagen treatment and gene editing approaches (Wang et al., 2014; Acevedo-Garcia et al., 2017). Notwithstanding, *Tamlo* alleles were more susceptible to *MoT* (Gruner et al., 2020).

It has been demonstrated that chemical and physical mutagens are able to induce nucleotide changes, including substitutions, insertion, or deletions (Ahloowalia and Maluszynski, 2001; Du et al., 2017; Krasileva et al., 2017; Ichida et al., 2019), that represent loss-of-function mutations resulting in favorable, fungal-resistant phenotypes (Acevedo-Garcia et al., 2017; Hussain et al., 2018). Targeting Induced Local Lesions IN Genomes (TILLING) is a reverse genetic approach to identify mutant allele (McCallum et al., 2000), and it has been used to discover mutant alleles in wheat, rice, barley and many other species. The target traits, such as wheat starch quality (Slade et al., 2005, 2012; Hazard et al., 2012), rice phytic acid and starch (Kim and Tai, 2014; Kim et al., 2018), have been improved through the approach. There are several different methods have been developed to TILL mutant alleles, such as gel electrophoresis based on enzyme digestion (Till et al., 2006), high resolution melting (Dong et al., 2009; Acanda et al., 2014), and the higher throughput TILLING by sequencing (Tsai et al., 2011).

Here, we tried to establish a new strategy aimed at identifying *MoT* resistance in wheat based on knowledge associated with rice blast resistance. Specifically, we took advantage of the close evolutionary relationship between *MoT* and *M. oryzae (MoO)*, BLASTed rice blast S orthologs in the wheat reference genome, and analyzed their functional domains. We then used EMS mutagen treatment and TILLING by sequencing in order to identify mutant single nucleotide polymorphisms (SNPs) in the M2 population that severely impact gene function and that might have the potential to enhance blast resistance in wheat. Our approach provides a new strategy to enhance the genetic diversity of wheat blast resistant germplasm.

## Materials and Methods

### Plant Materials

Wheat (*Triticum aestivum* L.) cultivar Jing411 and its EMS-induced M2 mutated population (Guo et al., 2017) were used to identify mutant alleles in target genes. Five biological replicates of wild type (WT) were used as reference.

A total of 2,300 M2 individuals were used for mutation screening. M1 plants were strictly self-crossed by bagging, and a single seed was harvested from each plant to develop the M2 population, leaves of each M2 individual plants were sampled to extract DNA. All samples were normalized to the same concentration (50 ng/μl) and placed in 96-well plates. A two dimensional pooling scheme was used following protocol of Till et al. (2006) with modification, the 12 samples in each column were pooled into one sample, and the eight samples in each row were pooled into another (Supplementary Figure 1), a total of 571 pooled samples were obtained. All pooling samples were then used for TILLING by sequencing.

The M3 mutants which were predicted to have severe impacts were used to validate variations, each mutant line was planted 20–40 seeds according to their total seed amount. The seeds were planted in the experimental field of Institute of Crop Sciences, Chinese Academy of Agricultural Sciences. Seedling leaf of each individual was sampled to extract DNA for mutation confirmation.

### Sequence Blast and Analysis

We used the DNA sequences of rice blast S genes *Os10g0548200*, *Os03g32230*, and *Os04g0401000*, from the Rice Annotation Project Database^1^, as templates to BLAST in the wheat reference genome Version 2.0^2^. The wheat orthologs found across the three sub-genomes were then analyzed in NCBI’s database^3^ in order to access their conserved functional domains, which were used as the target sequences of mutation detection by TILLING. Specific primers (Supplementary Table 1) were designed using the software GenoPlexs Primer Designer (Molbreeding Company, China), and used to amplify pooling samples.

### TILLING by Sequencing

The PCR reaction and library construction was prepared using the GenoPlexs Multiplex-PCR Library Prep Kit (Molbreeding Company, China), each step was performed according to the kit manual. The PCR reaction included 50 ng DNA, 1× T PCR Master Mix with improved high-fidelity pfu thermostable DNA polymerase and the primer mix. Amplification conditions included denaturation at 95°C for 5 min, followed by 32 cycles of 95°C for 30 s, 60°C for 30 s, and 72°C for 5 min on an ABI 9700 thermal cycler. The PCR products were then fragmented with an ultrasonic cleaner (Xinzhi Biotechnology, Ningbo, China, Scientz08-III) and, the fragment size and concentration were detected by agarose gel electrophoresis. After normalization, the products were purified with AMPure XP (Beckman Coulter, A63880).

The purified products were further used to add adaptor and barcode. Firstly the ends were repaired with Repair Enzyme by incubating 20 min on an ABI 9700 thermal cycler, and the A base was added to 3′ ends at the same time; then the adapters were added, which was incubated at 22°C for 60 min on an ABI 9700 thermal cycler. Then, a second purification round was followed before adding barcode. Finally, the barcode was added in conditions of denaturation at 98°C for 2 min, followed by 12 cycles of 98°C for 30 s, annealing for 30 s, and 72°C for 40 s, final extension at 72°C for 4 min. The sequence of barcode was AGTCGGAGGCCAAGCGGTCTTAGGAAGACAANNNNNN NNNNCAACTCCTTGGCTCACA, and the bottom adapter was TTGTCTTCCTAAGGAACGACATGGCTACGATCCGACT.

After a third purification round and fragment size detection, the library was sequenced by MGISEQ2000 (MGI Tech Co., Ltd., China).

### Mutation Detection

We filtered the raw reads to fetch clean reads using the software fastp V0.20.0 with parameters -n 10 -q 20 -u 40 (Chen et al., 2018). The Clean reads (BioProject ID PRJCA004347, deposited at National Genomics Data Center)^4^ were mapped to amplicon sequences of Chinese Spring (IWGSC RefSeq V1.0) using BWA-mem with default parameters^5^ (Li and Durbin, 2009). Sorting were performed with Picard (Version 2.1.1)^6^. GATK’s (version v3.5-0-g36282e4) module UnifiedGenotyper was used to call SNPs with parameters: -dcov 1000000 -minIndelFrac 0.15 -glm BOTH -l INFO; and module VariantFiltration was used to filter variants with parameters: -filterExpression “MQ0 ≥ 4 & ((MQ0/(1.0 ^∗^ DP)) > 0.1),” -filterName “HARD_TO_VALIDATE,” -filterExpression “DP < 5 | | QD < 2,” -filterName “LOW_READ_SUPPORT.” Variants were discovered from the VCF file (Supplementary File 1) using Perl scripts (Supplementary File 2). SNPs with <5× sequencing depth were treated as missing data. The variations between WT and Chinese Spring were filtered out.

The called SNPs were further corrected with frequency. All of the heterozygous sites in WT were considered to be false positive, and they were firstly filtered out before correction with ratio of alter alleles depth to read depth ≤0.20 or ≥0.80, which was higher than those of mutant call. Then SNPs were corrected in mutant pooling samples with the following threshold, when the ratio of alter alleles depth to read depth ≤0.05, the SNPs were considered to be homozygous and identity with reference sites, with the ratio ≥0.95 were considered to be homozygous mutation sites, and with the remainder being considered as heterozygous mutation sites. The mutant SNPs identified in both the row-pooling-sample and the line-pooling-sample were considered to represent true mutations, while those that were only detected in either the row-pooling-sample or the line-pooling-sample were considered to be false positives (Supplementary Figure 1 and Supplementary File 3). Those of SNPs identified in the antisense strands were substituted by complementary bases in the sense strands, and listed in tables.

The mutation density of each gene was calculated by dividing the total number of SNPs by the total sequenced length (sequenced length of the gene multiplied by number of sampled individuals).

### Prediction of Mutation Effects

The SNPs classified as true positives were then classified into promoter, exon and intron regions according to their respective location on the genes, and the effects on protein translation of those lying in the coding region analyzed. The impacts of missense mutations were predicted using the online software PROVEAN (Protein Variation Effect Analyzer)^7^.

### Structure Prediction of Mutant Proteins

The secondary protein structure was predicted using the website http://www.prabi.fr/. The three-dimensional (3D) structures of non-sense and missense mutations were predicted using the SWISS-MODEL server and 3D models generated from multiple threading alignments of amino acid sequences (Bordoli et al., 2009; Biasini et al., 2014). The protein structures were edited and visualized using the software Deepview/Swiss PDB Viewer V.4.1.0.

### Validation of Mutant Lines by Sanger Sequencing

Specific primers for each SNP were designed manually according to the specificity of 3′ end. The PCR reaction included 1× Taq Plus Master Mix II (Vazyme Biotech Co., Ltd.), 10 μm primer mix and 100 ng/μl genomic DNA. Amplification conditions included denaturation at 95°C for 3 min, followed by 35 cycles of 95°C for 15 s, annealing for 20 s, and 72°C for 1 min. The PCR products were then detected by 1% agarose gel electrophoresis, those with single band were further sequenced to detect the specificity of primers. Finally, individual samples of each mutant were amplified by the specific primers with two biological repeats, and sequenced by Sanger sequencing to validate SNP variation.

## Results

### Identification of Homologous Rice Blast S Genes in Wheat

Through BLAST in the wheat reference genome, orthologs of rice blast S gene *Bsr-k1* (*Os10g0548200*) were identified in the first homologous group 1A (TraesCS1A02G207700), 1B (TraesCS1B02G221400), and 1D (TraesCS1D02G211000) (Supplementary Figure 2A), while *Bsr-d1* (*Os03g32230*) orthologs were present on the seventh homologous group 7A (TraesCS7A02G160700), 7B (TraesCS7B02G065700), and 7D (TraesCS7D02G161800) (Supplementary Figure 2B). However, no *Pi-21* (Os04g0401000) orthologs were identified in the wheat reference genome (Supplementary Table 2).

**FIGURE 1:**
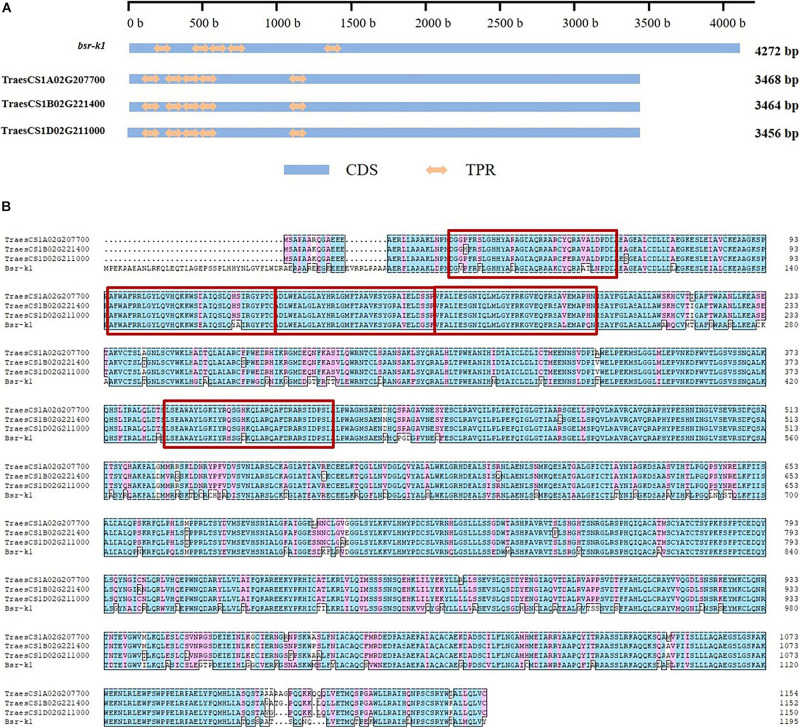
Conserved domains and amino acid sequence of gene *Bsr-k1* and its respective wheat orthologs. **(A)** conserved domains, **(B)** amino acid sequence. CDS means coding sequence, TPR means tetratricopeptide repeat, the TPRs are highlighted by red rectangles.

The three *Bsr-k1* wheat orthologs consist of 20 exons and 19 introns (Supplementary Figure 2A), and include five sets of conserved functional TPR domains that were observed in wheat homologous genes (Figure 1). Their respective observed protein sequence identity was higher than 97%, and more than 80% when compared to BSR-K1.

**FIGURE 2:**
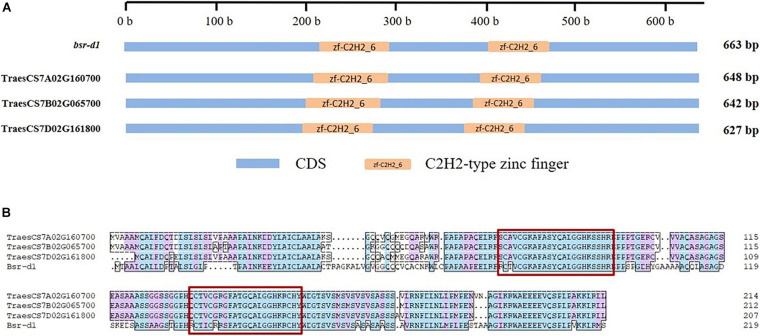
Conserved domains and amino acid sequence of gene *Bsr-d1* and its respective wheat orthologs. **(A)** conserved domains, **(B)** amino acid sequence. CDS means coding sequence, the amino acid sequence of two conserved zinc fingers are highlighted by red rectangles.

We have also observed that the sequence identity of BSR-D1 with its wheat orthologs was only 62.3–64.1%. However, the C2H2-type zinc finger domains of *Bsr-d1* were highly conserved in three wheat orthologs (Figure 2), whereby its function might be maximally preserved in wheat.

### Mutation Density and Substitution Types of Target Fragments

The density of mutations in the six target fragments ranged from 1/1107.1 to 1/230.7 kb (Table 1), with an average of 1/309.5 kb. The lowest mutation density was found in the gene TraesCS7A01G160700, where only three mutants were detected.

**TABLE 1:** Mutation densities of *Bsr-k1* and *Bsr-d1* wheat orthologs in the M2 population after EMS treatment.

Gene	NCBI accession number	Gene size (kb)	Sequenced fragment size (kb)	Mutation number	Mutation density
TraesCS1A02G207700	MW388661	10.948	6.950	55	1/290.6 kb
TraesCS1B02G221400	MW388662	8.444	4.514	45	1/230.7 kb
TraesCS1D02G211000	MW388663	8.260	4.884	46	1/244.2 kb
TraesCS7A02G160700	MW388664	1.163	1.444	3	1/1107.1 kb
TraesCS7B02G065700	MW388665	0.904	1.469	10	1/337.9 kb
TraesCS7D02G161800	MW388666	0.905	1.427	12	1/273.5 kb

More than 90% of base substitutions detected were transitions, and the remainder were transversions. All transversions occurred in intronic regions and corresponded to mutations from C, T, or A into G, A, or C. The only exception was found in the 5′UTR region and included a C > G transversion that resulted in a start-codon gain in line E1354. No deletions or insertions were detected in the population.

### The Effects of SNPs in *Bsr-k1* Wheat Orthologs

**FIGURE 3:**
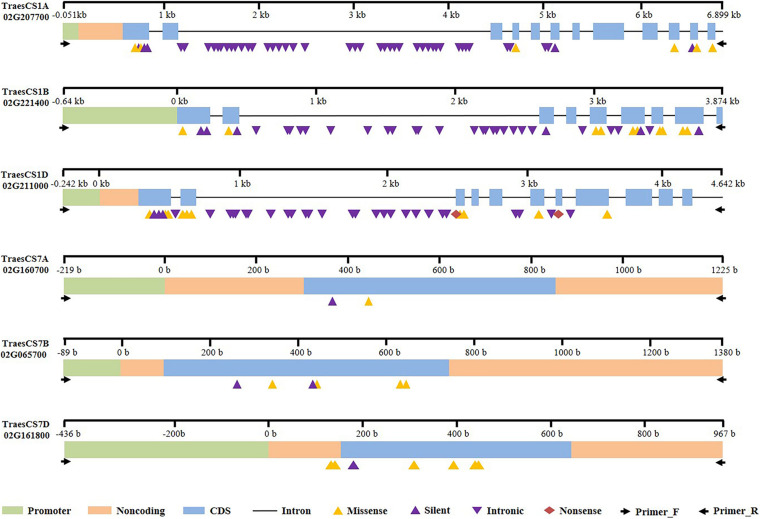
SNP distribution in the six target fragments of *Bsr-k1* and *Bsr-d1* wheat orthologs.

The PCR amplicons were verified by agarose gel electrophoresis (Supplementary Figure 3 and not shown) and, after fragmentation, purification and adding adaptor, the products were sequenced by next-generation sequencing, and the sequences of WT were submitted to National Center for Biotechnology Information (NCBI) database (Table 1). In total, we identified 146 mutated SNPs in the three *Bsr-k1* wheat orthologs in total in the M2 population. The mutations were distributed across the promoter, exonic and intronic regions (Figure 3). The mutations overlapping the coding region (CDS) were classified into silent, missense and non-sense mutation types due to their respective effects on amino acid translation. A total of 10 mutants were predicted to have severe effects on gene function.

Moreover, a total of 55 SNPs in the gene TraesCS1A02G207700 were identified, including 15 and 40 SNPs located in exons and introns, respectively (Table 2, Figure 3, and Supplementary Table 3). Among the 11 SNPs found in the CDS region, five resulted in missense mutations and two (line E758 and E325) were predicted to severely impact gene function, while the other six represented silent mutations. We also found a start-codon-gain mutant in the 5′UTR region, which resulted in a 132-base advance of the starting codon without any downstream frameshift.

**TABLE 2:** SNPs identified in *Bsr-k1* wheat orthologs and their predicted impact on protein function.

Line	Region	Allele^*a*^	Mutation Type	Variation in Amino Acid^*b*^	PROVEAN Score	Prediction
**TraesCS1A02G207700**						
A32	5′UTR	C121T				
E1354	5′UTR	C159G	start codon gained			
A408	5′UTR	C196T				
A410	5′UTR	G254A				
E333	CDS1	C301T	Missense	P4S	0.788	Neutral
E038-9	CDS1	C342T	Silent	L17=		
E049-1	CDS1	G346A	Missense	A19T	−0.433	Neutral
E054-10	CDS1	C399T	Silent	H36=		
E439	CDS1	C483T	Silent	A65=		
E758	CDS4	C5070T	Missense	A149V	−3.825	Deleterious
E203	CDS6	G5413A	Silent	Q188=		
E325	CDS10	G6403A	Missense	A407T	−3.28	Deleterious
E1258	CDS11	C6629T	Silent	C448=		
E049-1	CDS11	G6641A	Silent	V452=		
E630	CDS12	G6807A	Missense	G472D	1.357	Neutral
**TraesCS1B02G221400**						
A53	promoter	G-429A				
E48	promoter	C-212T				
E046-3	promoter	C-192T				
E041-12	promoter	C-84T				
E1180	CDS1	C11T	Missense	P4I	0.108	Neutral
E833	CDS1	G87A	Silent	G29=		
E1015	CDS1	C162T	Silent	A54=		
E038-16	CDS2	G353A	Missense	G76E	−4.464	Deleterious
E607	CDS2	C438T	Silent	Y104=		
E889	CDS3	G2567A	Silent	Q117=		
E1272	CDS5	G2885A	Missense	S161R	−1.465	Neutral
E786	CDS5	C2888T	Missense	S162F	−3.81	Deleterious
E536	CDS6	G3113A	Missense	E192K	−0.464	Neutral
E1294	CDS6	G3195A	Missense	G219E	−3.792	Deleterious
E1171	CDS6	G3196A	Silent	G219=		
E1418	CDS7	G3305A	Missense	E230K	−1.289	Neutral
E410	CDS7	G3308A	Missense	A231T	−2.888	Deleterious
E148	CDS8	G3506A	Missense	D267N	−0.167	Neutral
E601	CDS8	G3522A	Missense	R272K	0.184	Neutral
E118	CDS8	C3625T	Silent	R306=		
E496	CDS8	C3625T	Silent	R306=		
**TraesCS1D02G211000**						
E653	5′UTR	C5T				
E1344	5′UTR	C39T				
E028-15 (II)	5′UTR	C83T				
E044-9	5′UTR	C109T				
E136	5′UTR	G203A				
A316	CDS1	C242T	Missense	A3V	−0.649	Neutral
E042-1	CDS1	C246T	Silent	P4=		
E1151	CDS1	C333T	Silent	S33=		
E316	CDS1	C342T	Silent	H36=		
E1184	CDS1	C356T	Missense	A41V	0.926	Neutral
E972	CDS1	G380A	Missense	R49K	0.625	Neutral
E1180	CDS2	G547A	Missense	A74T	−0.034	Neutral
E91	CDS2	C604T	Missense	P93S	−3.459	Deleterious
E958	CDS2	G626A	Missense	R100Q	−1.297	Neutral
E60	CDS3	C2686T	Non-sense	Q109stop	−6.915	Deleterious
E203	CDS3	G2701A	Missense	D114N	1.075	Neutral
E315	CDS3	C2740T	Missense	P127S	−5.116	Deleterious
E054-9	CDS6	G3275A	Missense	E192K	−0.597	Neutral
E724	CDS7	G3525A	Non-sense	W249stop	−16.106	Deleterious
E539	CDS8	C3849T	Missense	T327I	−1.544	Neutral

*^*a*^: Start from the initiation site of the gene.*

*^*b*^: “=” means Synonymous change.*

A total of 45 mutated SNPs distributed across promoter, exonic, and intronic regions were identified in the gene TraesCS1B02G221400 (Table 2, Supplementary Table 3, and Figure 3). Among these, we found 10 missense mutations, 4 of which were predicted to have a deleterious impact. Furthermore, there were four mutations in the promoter region that may also lead to variations in gene function.

Finally, we identified 20 SNPs in the exonic regions of the gene TraesCS1D02G211000 (Table 2, Supplementary Table 3, and Figure 3), of which two were non-sense and 10 missense mutations. Importantly, the two stop-gained mutants (E60 and E724) as well as C604T (E91) and C2740T (E315) might lead to severe impacts on function.

### The Effects of SNPs in *Bsr-d1* Wheat Orthologs

A total of 25 SNPs were found in the three wheat orthologs of *Bsr-d1* in the M2 population, including one start-codon loss, 10 missense mutations, and several others located in the UTR and promoter regions (Table 3). PROVEAN analysis predicted that the loss of the start-codon (G135A) is neutral due to the existence of an alternative start codon within 12 base pairs without any downstream frameshift. This analysis also predicted that the C488T mutation in TraesCS7A02G160700 and the C304T and G497A mutations in TraesCS7D02G161800 have a severe effect on protein function.

**TABLE 3:** SNPs identified in *Bsr-d1* wheat orthologs and their predicted impact on protein function.

Line	Region	Allele^*a*^	Mutation Type	Variation in Amino Acid^*b*^	PROVEAN score	Prediction
**TraesCS7A02G160700**						
E038-14	5′UTR	G217A				
E035-7	CDS1	C412T	Silent	S16=		
E038-6	CDS1	C488T	Missense	L42F	−4	Deleterious
**TraesCS7B02G065700**						
E049-4	5′UTR	C38T				
A305	CDS1	C220T	Silent	D32=		
E051-2	CDS1	G314A	Missense	A64T	−1.002	Neutral
A146	CDS1	G466A	Silent	G114=		
E1300	CDS1	G470A	Missense	E116K	−0.675	Neutral
A17	CDS1	G620A	Missense	V166M	−0.445	Neutral
E035-13	CDS1	C633T	Missense	A170V	−1.283	Neutral
E053-12	3′UTR	C859T				
E023-10	3′UTR	C1202T				
E024-11	3′UTR	G1235A				
**TraesCS7D02G161800**						
A42	promoter	G-370A				
A196	promoter	C-361T				
A259	promoter	C-258T				
A417	promoter	G-236A				
E024-3	promoter	G-143A				
E054-8	CDS1	G135A	start codon lost	M1I	−0.584	Neutral
E040-14	CDS1	C197T	Missense	P22L	−1.62	Neutral
A277	CDS1	C246T	Silent	A38=		
E044-3	CDS1	C304T	Missense	P58S	−2.879	Deleterious
A34	CDS1	C421T	Missense	R97W	−0.816	Neutral
E149	CDS1	G491A	Missense	S119D	−1.235	Neutral
E044-2	CDS1	G497A	Missense	G122D	−3.815	Deleterious

*^*a*^: Start from the initiation site of the gene.*

*^*b*^: “=” means Synonymous change.*

### Verification of SNPs With Severe Impacts in M3

A total of 13 SNPs with severe impacts were discovered in the six target genes, 12 of them and five of those located in UTR and promoter region were further validated, except mutant line E044-3 because of insufficient seeds. A total of 12 sets of specific primers were used after electrophoresis and sequencing evaluation (Supplementary Table 4 and Supplementary Figure 4). 100% of the SNPs were confirmed by Sanger sequencing, and all of the SNPs in M3 were consistent with those from pooled M2 population (Table 4).

**TABLE 4:** Validation of SNPs in M3 generation.

Gene	Mutant	Allele	Total Number of tested individuals	Mutants	Non-mutants
TraesCS1A02G207700	E758	C5070T	35	34	1
	E325	G6403A	30	30	0
TraesCS1B02G221400	E038-16	G353A	18	15	3
	E786	C2888T	27	26	1
	E1294	G3195A	27	27	0
	E410	G3308A	32	32	0
TraesCS1D02G211000	E91	C604T	30	26	4
	E60	C2686T	16	16	0
	E315	C2740T	34	33	1
	E724	G3525A	17	14	3
TraesCS7A02G160700	E038-14	G217A	37	36	1
	E038-6	C488T	30	30	0
TraesCS7D02G161800	A42	G-370A	24	23	1
	A196	C-361T	24	8	16
	A259	C-258T	24	17	7
	E024-3	G-143A	33	32	1
	E044-3	C304T	31	18	13

### Secondary and 3D Structure Variation of Target Proteins

**FIGURE 4:**
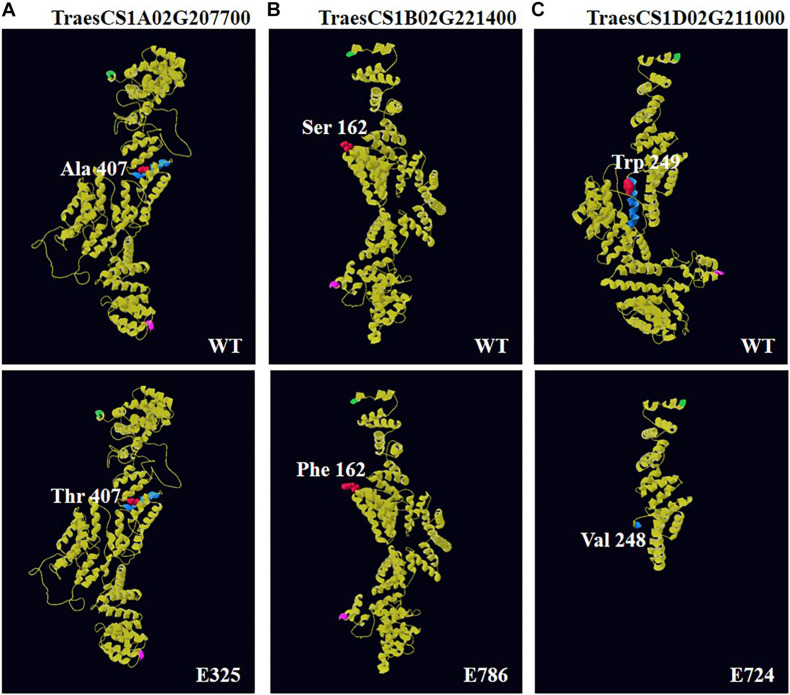
Three-dimensional (3D) models of BSR-K1 wheat orthologs and mutants. The models were constructed using template 4buj.2.B, a *S. cerevisiae* Ski2-3-8 complex. **(A)** 3D structure of TraesCS1A02G207700 and its mutant E325; **(B)** 3D structure of TraesCS1B02G221400 and its mutant E786; **(C)** 3D structure of TraesCS1D02G211000 and its mutant E724. The N-terminal is highlighted in green, the C-terminal in pink, and the residue immediately before and after each mutation is shown in red, its secondary structure in blue.

Using online software, the predicted three-dimensional protein models of BSR-K1 wheat orthologs showed homology to the *Saccharomyces cerevisiae* Ski2-3-8 complex with multiple alpha helices (Figure 4, Supplementary Figure 5, and Supplementary Table 5). The amino acid change Ala407Thr in line E325, located in the fifth TPR region, which was a change from hydrophobic residue to hydrophilic residue and, resulted in the formation of a random coil instead of an alpha helix (Supplementary Figure 5A and Supplementary Table 5). On the contrary, the mutation Ser162Phe in line E786, hydrophilic residue to hydrophobic Phe residue, resulted in a reduced random coil that enabled more residues to form an alpha helix and less to participate in an extended strand (Supplementary Figure 5B and Supplementary Table 5). The truncation mutation found in line E724 led to the loss of the fifth TPR region and remaining residues (Figure 4C and Supplementary Table 5), which might significantly affect protein function.

## Discussion

### Our Mutated Population Resulted in the Discovery of Multiple Mutations in One Line and the Same SNP in Multiple Lines

Mutational types and their frequency are often correlated with the mutagens and the species where they occur. Based on high-throughput data from exome capture and whole-genome sequencing, transitions generally represent over 90% of EMS treatment induced mutations (Henry et al., 2014; Krasileva et al., 2017), compared to just ∼40–50% using heavy ion beams and fast neutrons (Li G.T. et al., 2017; Ichida et al., 2019). In our study, the proportion of transition mutations observed were 91 and 98% in the genome and exon/promoter regions, respectively, which is consistent with previously reported EMS-induction results (Henry et al., 2014; Krasileva et al., 2017). While an individual mutant line can carry thousands of mutated alleles (Krasileva et al., 2017; Li G.T. et al., 2017; Hussain et al., 2018), we found that 12 out of 175 lines carried more than one mutated SNPs (specifically two mutations in each line). Most of them in our experiment were either located in intronic regions, represented silent mutations or had neutral effects. Only lines E48 and A53 carried mutations in the promoter region of TraesCS1B02G221400 that might affect gene function, no loss-of-function double mutant line of the target genes was directly created in the current M2 population. These results confirmed that multiple mutations existed in one individual line.

In addition, a previous study focusing on mutated tetraploid and hexaploid wheat populations, identified 1.4 out of 10 million SNPs (i.e., around 14%) in more than one individual line through exome capture (Krasileva et al., 2017). In this study, we uncovered 8 SNPs (5%) in 2–5 lines, most of which are located in intronic regions, probably due to the lesser constraint affecting intron evolution. These results demonstrated that the same SNP can be found in multi-individuals of the same mutated population even if through EMS treatment. As mentioned above, the EMS mutagen induces transition-type mutations such as G > A and C > T, transversions are thought to represent non-EMS mutations and instead result from genetic heterogeneity or sequencing errors associated with lower coverage (King et al., 2015; Krasileva et al., 2017), whereas it has been reported that transversions in different species induced by chemical mutagens including EMS were presented with lower percentage (Spencer-Lopes et al., 2018). In our study, five out of eight mutations were non-EMS type. Since we have excluded positions with less than 5× depth, it is unlikely that these mutations result from sequencing error. In addition, seeds used for EMS treatment and WT were derived from the same branch, so the probability of genetic heterogeneity is very low. Taken together, these transversions probably derived from EMS treatment.

### Using Rice Blast Susceptible Genes Opens a New Window to Promote Wheat Blast Resistance Breeding Through Mutation Induction

The promoter region controls the transcription of genes through the binding of specific transcription factors. Accordingly, variations in the genomic sequence of both transcription factors and promoter might alter gene function. *WRKY76* is a transcription factor that binds to W-box elements and its overexpression results in decreased resistance to rice blast (Yokotani et al., 2013). At the same time, a SNP in the promoter region (-618) and consequent *bsr-d1* knockout leads to an increased binding affinity with the transcription factor MYBS1, which, in turn, enhances blast resistance (Li W. et al., 2017). The *bsr-d1* wheat orthologs reported here maintained the C2H2-type zinc finger functional domain, and we report mutations in the promoter and coding regions of the gene that have the potential to enhance *MoT* resistance.

A majority of disease resistant genes encode for conservative proteins containing a nucleotide binding site with leucine rich repeats. In contrast, while TPR mediate an alternative immune response mechanism in plants, the loss-of-function BSR-K1 TPR protein is unable to bind to the mRNA of the *OsPAL* gene family, resulting in blast resistance in rice (Zhou et al., 2018). We found that the BSR-K1 TPR protein is highly conserved in wheat with over 80% sequence identity. Moreover, we identified five tandem repeats, multiple truncation and missense mutations with deleterious effects in the three sub-genomes that lead to the destruction of the TPR domain in a similar fashion to what is observed in rice. The susceptible powdery mildew gene *Mlo* found in barley is conserved across plant species (Kusch et al., 2016), and its loss-of-function mutation in wheat and other species leads to enhanced powdery mildew disease resistance (Acevedo-Garcia et al., 2017). The mutants identified in this study might also provide enhanced immunity and resistance to wheat blast. Although the resistance level needs to be validated under infected-field conditions, these alleles have not been previously reported in the literature, and might represent a valuable new resource for wheat blast (or even other fungi) disease resistance breeding.

As an hexaploid species, a mutation on one of the three sub-genomes may or may not lead to phenotypic variation in wheat. Hence, it is necessary to pyramide the three homologs before evaluating resistance, and it would be particularly beneficial to pyramide the deleterious mutations reported in the five genes mentioned above in order to evaluate their interactions against *MoT* and other fungal diseases.

## Conclusion

We obtained six wheat orthologs of two rice blast susceptible genes through homologous gene comparison and identified loss-of-function mutations in these genes in a M2 population. We discovered that 13 mutant alleles have deleterious effects and might enhance wheat blast resistance. Our research provides a new strategy and novel gene resources to tackle disease resistant wheat breeding.

## Data Availability Statement

The raw data supporting the conclusions of this article will be made available by the authors, without undue reservation.

## Author Contributions

HG and LL designed the experiments. HG and QD analyzed the data, prepared all tables and figures, and wrote the manuscript with input from all co-authors. HG, QD, YX, HX, LZ, JG, SZ, XS, and TI performed the experiments. All authors contributed to the article and approved the submitted version.

## Conflict of Interest

The authors declare that the research was conducted in the absence of any commercial or financial relationships that could be construed as a potential conflict of interest.

## Footnotes

1 https://rapdb.dna.affrc.go.jp

2 https://wheat-urgi.versailles.inra.fr/

3 https://www.ncbi.nlm.nih.gov/Structure/cdd/wrpsb.cgi

4 https://bigd.big.ac.cn/gsub/

5 http://bio-bwa.sourceforge.net/bwa.shtml

6 http://broadinstitute.github.io/picard/

7 http://provean.jcvi.org/index.php

## Supplementary Material

Supplementary Figure 1 Diagram showing the sample pooling procedure on a 96-well-plate. The 12 samples corresponding to the gray line C were pooled into a single line-pooling-sample, while the eight samples in the blue row seven were pooled into a separate row-pooling-sample. The green sample detected in both line- and row-pooling-samples was considered a positive mutant, whereas the pink sample only detected in the row-pooling-samples was considered a negative mutant.

Supplementary Figure 2 Structure of orthologous genes of **(A)** *Bsr-k1* (located on chromosomes 1A, 1B, and 1C) and **(B)** *Bsr-d1* (located on chromosomes 7A, 7B, and 7D).

Supplementary Figure 3 Verification of the amplicons of gene TraesCS1A02G207700 by agarose gel electrophoresis. Forty-eight samples (E1–H12) from one of the 96-well pooling plates. M, DNA marker with fragment size of 15,000, 8,000, 6,000, 4,000, 3,000, 2,000, 1,000, and 500 bp.

Supplementary Figure 4 An example of amplification using specific primers in M3 mutant lines of E325, E786, and E60 by agarose gel electrophoresis. E325-1, E325-2 mean the different M3 individuals of mutant E325, and so on down the gel.

Supplementary Figure 5 Secondary structure of BSR-K1 wheat orthologs and mutants. **(A)** WT and mutant E325 of TraesCS1A02G207700; **(B)** WT and mutant E786 of TraesCS1B02G221400; **(C)** WT and mutant E724 of TraesCS1D02G211000. The mutant residue is highlighted by black triangle, and the variation secondary structure is highlighted in red rectangle.
