# Supplementary figures and images for: Identification of Rice Blast Loss-of-Function Mutant Alleles in the Wheat Genome as a New Strategy for Wheat Blast Resistance Breeding
Source: Front Genet. 2021 May 19;12:623419. doi: 10.3389/fgene.2021.623419 (PMC8170139; doi:10.3389/fgene.2021.623419)

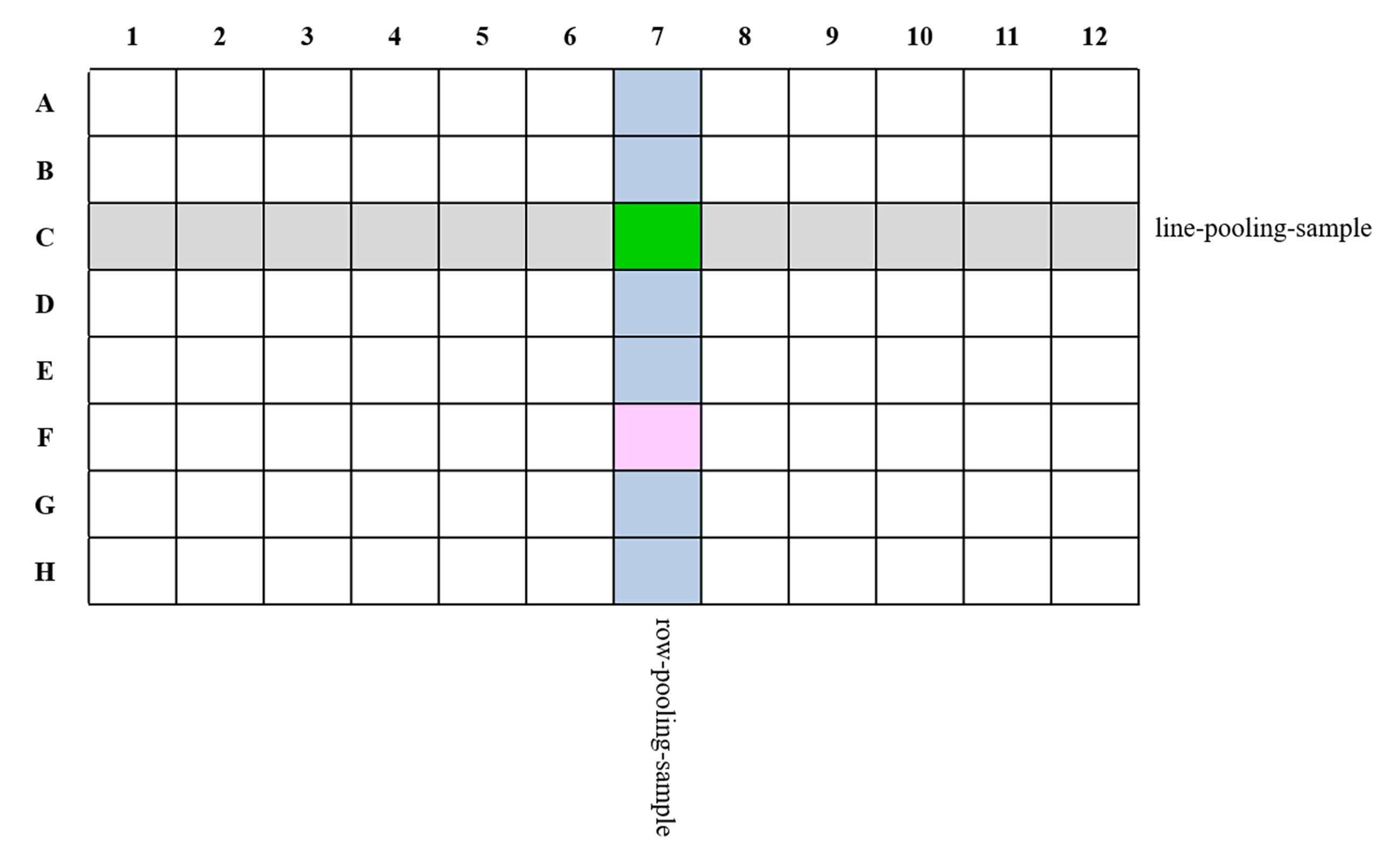

Supplement: Supplementary Figure 1 — Diagram showing the sample pooling procedure on a 96-well-plate. The 12 samples corresponding to the gray line C were pooled into a single line-pooling-sample, while the eight samples in the blue row seven were pooled into a separate row-pooling-sample. The green sample detected in both line- and row-pooling-samples was considered a positive mutant, whereas the pink sample only detected in the row-pooling-samples was considered a negative mutant. [file Image_1.JPEG]

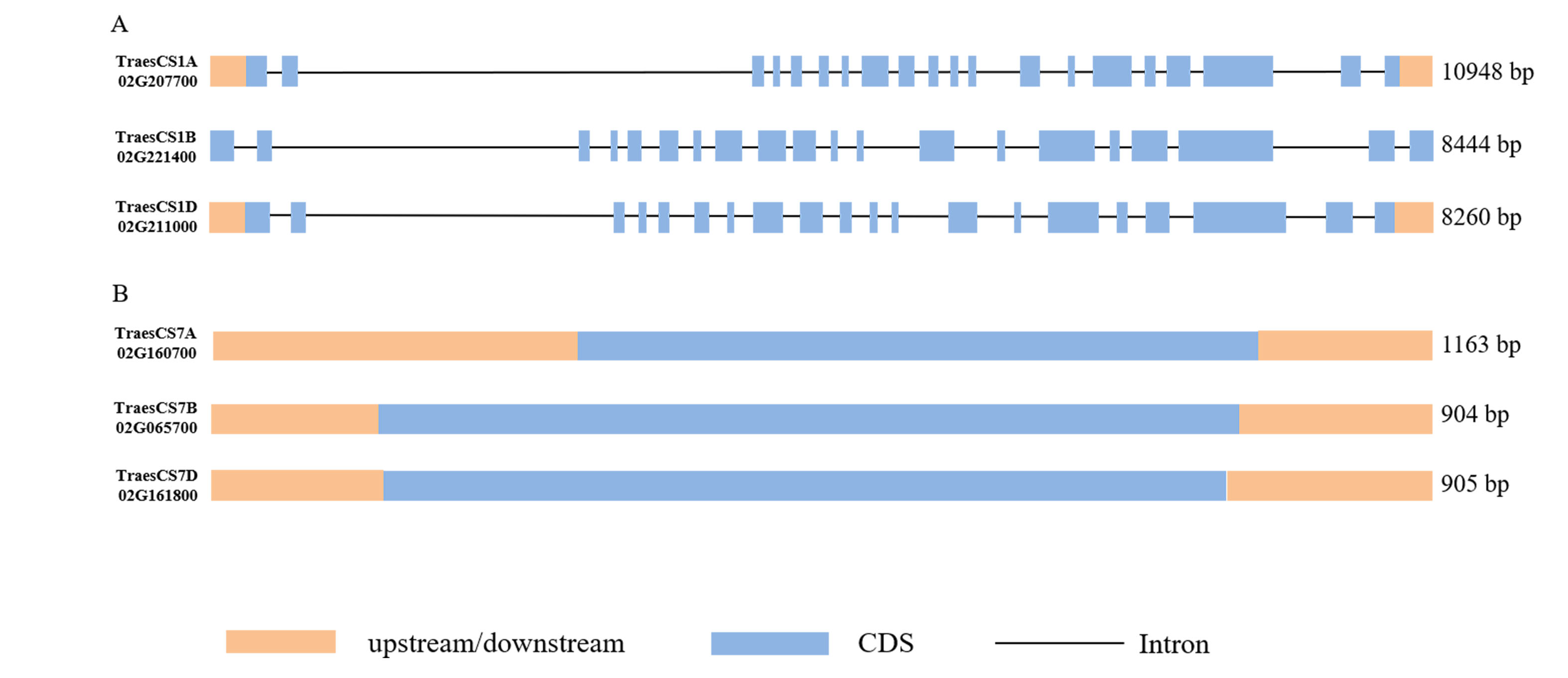

Supplement: Supplementary Figure 2 — Structure of orthologous genes of (A) Bsr-k1 (located on chromosomes 1A, 1B, and 1C) and (B) Bsr-d1 (located on chromosomes 7A, 7B, and 7D). [file Image_2.JPEG]

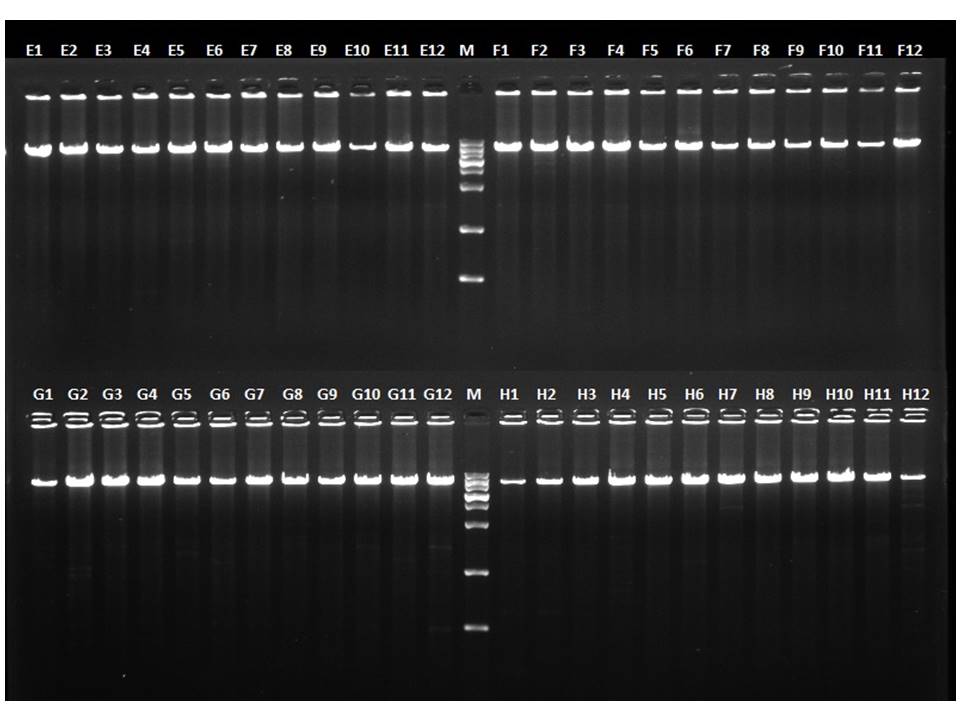

Supplement: Supplementary Figure 3 — Verification of the amplicons of gene TraesCS1A02G207700 by agarose gel electrophoresis. Forty-eight samples (E1–H12) from one of the 96-well pooling plates. M, DNA marker with fragment size of 15,000, 8,000, 6,000, 4,000, 3,000, 2,000, 1,000, and 500 bp. [file Image_3.JPEG]

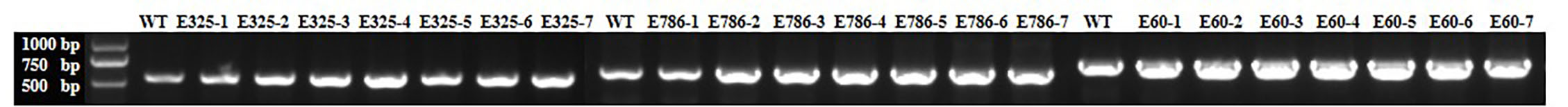

Supplement: Supplementary Figure 4 — An example of amplification using specific primers in M3 mutant lines of E325, E786, and E60 by agarose gel electrophoresis. E325-1, E325-2 mean the different M3 individuals of mutant E325, and so on down the gel. [file Image_4.JPEG]

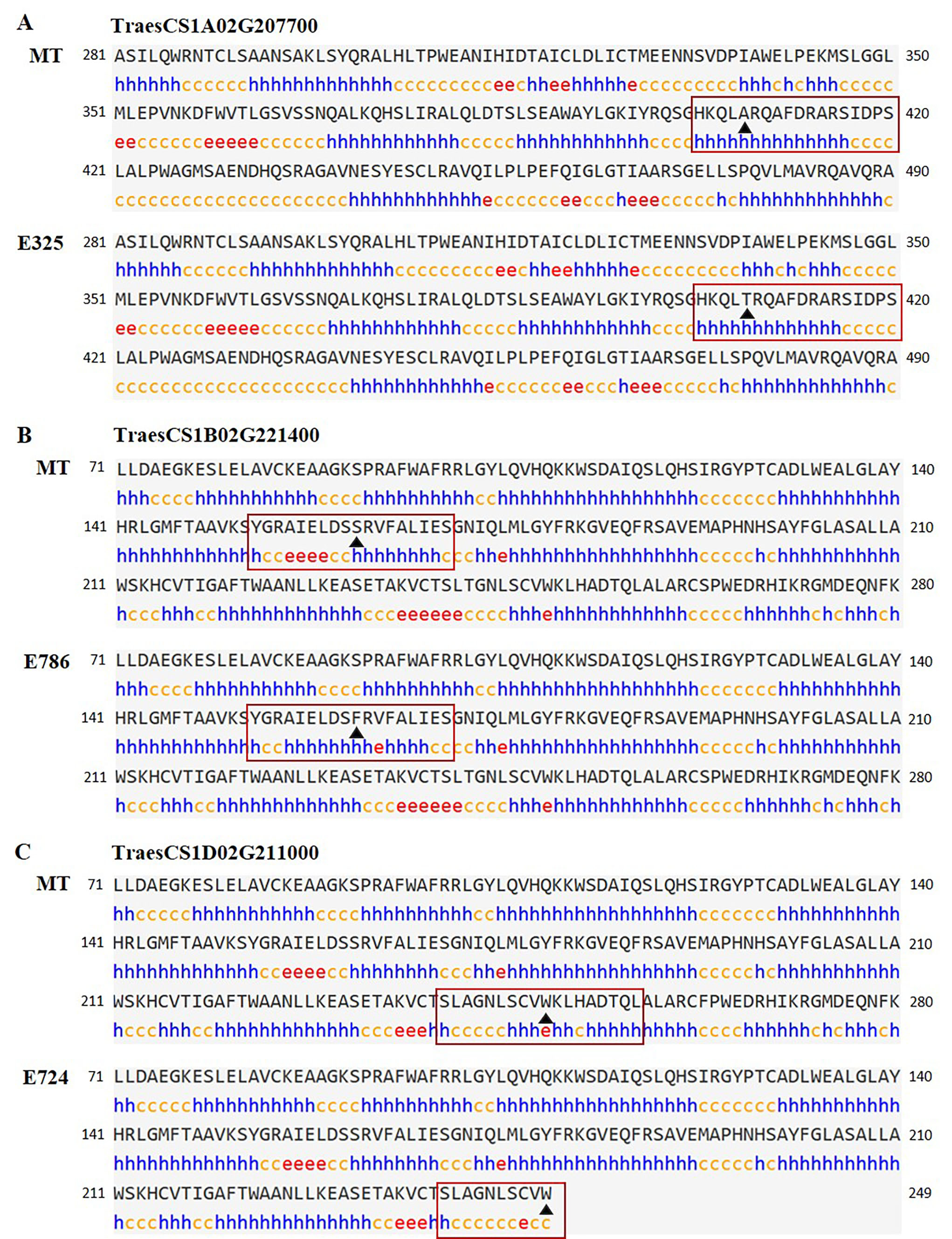

Supplement: Supplementary Figure 5 — Secondary structure of BSR-K1 wheat orthologs and mutants. (A) WT and mutant E325 of TraesCS1A02G207700; (B) WT and mutant E786 of TraesCS1B02G221400; (C) WT and mutant E724 of TraesCS1D02G211000. The mutant residue is highlighted by black triangle, and the variation secondary structure is highlighted in red rectangle. [file Image_5.JPEG]
