# Supplementary material for: Identification of Rice Blast Loss-of-Function Mutant Alleles in the Wheat Genome as a New Strategy for Wheat Blast Resistance Breeding
Source: Front Genet. 2021 May 19;12:623419. doi: 10.3389/fgene.2021.623419 (PMC8170139; doi:10.3389/fgene.2021.623419)
Supplement: Supplementary file 8 [file Data_Sheet_3.docx]

#!usr/bin/perl -w

use File::Basename;

my $dir=shift;

my $prefix=shift;

my $depth_cut=shift;

my $hetfre1=shift;

my $hetfre2=shift;

my (%depth,%ref,%sample,%alt,%flag);

my @file=`ls $dir/*/*$prefix`;

my %position;

foreach my $vcf_file(@file)

{

chomp $vcf_file;

open IN,$vcf_file or die $!;

while (<IN>)

{

chomp;

next if ($_=~/^#/);

my @a=split /\t/,$_;

my $len1=length $a[3];

my $len2=length $a[4];

if ($a[4] =~/,/)

{

my $pos;

my @type=split /,/,$a[4];

my $l1=length $type[0];

my $l2=length $type[1];

my @seq;

if ($len1 ==1)

{

$position{"$a[0]\t$a[1]"}=1;

}

else

{

my $pos2=$a[1]+$len1-1;

my $tmp=substr ($a[3],1);

my $pos1=$a[1]+1;

$position{"$a[0]\t$pos1"}=1;

}

}

else

{

if ($len1 eq "1" && $len2 eq "1")

{

$position{"$a[0]\t$a[1]"}=1;

}

elsif ($len1 eq "1" && $len2 >1 )

{

$position{"$a[0]\t$a[1]"}=1;

}

elsif($len1 >1 && $len2 eq "1")

{

my $pos1=$a[1]+1;

my $pos2=$a[1]+$len1-1;

$position{"$a[0]\t$pos1"}=1;

}

}

}

close IN;

}

my %alt_type;

foreach my $vcf_file(@file)

{

chomp $vcf_file;

my $name=basename $vcf_file;

my @name=split /\./,$name;

$sample{$name[0]}=1;

my $depth_file=$vcf_file;

$depth_file=~s/filt\.bed.vcf/depth/g;

open IN,$depth_file or die $!;

while (<IN>)

{

chomp;

my @a=split /\t/,$_;

if ($a[2] >= $depth_cut)

{

if (exists $position{"$a[0]\t$a[1]"})

{

$depth{"$a[0]\t$a[1]"}{$name[0]}=$a[2];

}

}

#$ref{"$a[0]\t$a[1]"}=$a[2];

}

close IN;

open IN,$vcf_file or die $!;

while (<IN>)

{

chomp;

next if ($_=~/^#/);

my @a=split /\t/,$_;

my $genetype;

my $len1=length $a[3];

my $len2=length $a[4];

if ($a[4] =~/,/)

{

my $pos;

my @type=split /,/,$a[4];

my $l1=length $type[0];

my $l2=length $type[1];

my @seq;

my $flag_tmp=0;

if ($len1 ==1)

{

$ref{"$a[0]\t$a[1]"}{$a[1]}=$a[3];

push @seq,$a[3];

$pos =$a[1];

if ($l1 >1)

{

my $tmp=substr ($type[0],1);

push @seq,"ins$tmp";

}

else

{

push @seq,$type[0];

}

if ($l2>1)

{

my $tmp=substr ($type[1],1);

push @seq,"ins$tmp";

}

else

{

push @seq,$type[1];

}

}

else

{

next if ($l1 == 1 || $l2 ==1);

my $pos2=$a[1]+$len1-1;

my $tmp=substr ($a[3],1);

my $pos1=$a[1]+1;

$ref{"$a[0]\t$pos1"}{$pos2}=$tmp;

push @seq,$a[3];

if ($l1>$len1)

{

my $tmp=substr($type[0],$len1);

push @seq ,"ins$tmp";

}

else

{

my $tmp1=substr ($a[3],$l1);

push @seq,"del$tmp1";

$flag_tmp++;

}

if ($l2>$len1)

{

my $tmp=substr($type[1],$len1);

push @seq ,"ins$tmp";

}

else

{

my $tmp2=substr ($a[3],$l2);

push @seq,"del$tmp2";

$flag_tmp++;

}

$pos=$a[1]+1;

}

if ($a[-1] =~/(\d)\/(\d):(\d+),(\d+),(\d+):(\d+)/)

{

my $rate=sprintf "%.2f" ,($4+$5)/($6+0.0001);

my $t;

my $d_ref=$3;

my $d_alt="$4/$5";

my $t1=$1;

my $t2=$2;

if ($seq[$t1]=~/ins/ || $seq[$t1] =~/del/ || $seq[$t2] =~/ins/ || $seq[$t2] =~/del/)

{

$t="$seq[$t1]/$seq[$t2]";

}

else

{

$t="$seq[$t1]$seq[$t2]";

}

if ($flag_tmp==2)

{

$flag{"$a[0]\t$pos"}{$pos}="del";

}

else

{

$flag{"$a[0]\t$pos"}{$pos}="undefined";

}

$ref{"$a[0]\t$pos"}{$pos}=$seq[0];

$alt{"$a[0]\t$pos"}{$pos}{$name[0]}="$t\t$d_ref/$d_alt\t$rate";

$alt_type{"$a[0]\t$pos"}{$pos}{$t}=1;

}

}

else

{

my ($ref_d,$alt_d);

if ($a[-1] =~/(\d)\/(\d):(\d+),(\d+):(\d+)/)

{

$ref_d=$3;

$alt_d=$4;

}

my $rate=sprintf "%.2f" ,$alt_d/($ref_d+$alt_d);

my $d=$ref_d+$alt_d;

next if ($d <$depth_cut);

if ($len1 eq "1" && $len2 eq "1")

{

$ref{"$a[0]\t$a[1]"}{$a[1]}=$a[3];

if ($a[-1]=~/(\d)\/(\d):(\d+),(\d+):(\d+)/)

{

my $r=sprintf "%.2f" ,$4/($3+$4);

if ($r >= $hetfre2)

{

$genetype="$a[4]$a[4]";

}

elsif ($r<= $hetfre1 )

{

$genetype="$a[3]$a[3]";

}

else

{

$genetype="$a[3]$a[4]";

}

}

$alt{"$a[0]\t$a[1]"}{$a[1]}{$name[0]}="$genetype\t$ref_d/$alt_d\t$rate";

$flag{"$a[0]\t$a[1]"}{$a[1]}="snp";

$alt_type{"$a[0]\t$a[1]"}{$a[1]}{$a[4]}=1;

}

elsif ($len1 eq "1" && $len2 >1 )

{

$ref{"$a[0]\t$a[1]"}{$a[1]}=$a[3];

my $tmp=substr ($a[4],1);

if ($rate>= $hetfre2)

{

$genetype="ins$tmp";

}

elsif ($rate<= $hetfre1)

{

$genetype="$a[3]$a[3]";

}

else

{

$genetype="$a[3]/ins$tmp";

}

$alt{"$a[0]\t$a[1]"}{$a[1]}{$name[0]}="$genetype\t$ref_d/$alt_d\t$rate";

$flag{"$a[0]\t$a[1]"}{$a[1]}="ins";

$alt_type{"$a[0]\t$a[1]"}{$a[1]}{"ins$tmp"}=1;

}

elsif($len1 >1 && $len2 eq "1")

{

my $pos1=$a[1]+1;

my $pos2=$a[1]+$len1-1;

my $tmp=substr ($a[3],1);

$ref{"$a[0]\t$pos1"}{$pos2}=$tmp;

if ($rate>= $hetfre2)

{

$genetype="del$tmp";

}

elsif ($rate<= $hetfre1)

{

$genetype="$tmp$tmp";

}

else

{

$genetype="$tmp/del$tmp";

}

$flag{"$a[0]\t$pos1"}{$pos2}="del";

$alt{"$a[0]\t$pos1"}{$pos2}{$name[0]}="$genetype\t$ref_d/$alt_d\t$rate";

$alt_type{"$a[0]\t$a[1]"}{$a[1]}{"del$tmp"}=1;

}

}

}

close IN;

}

print "chr\tstart\tend\tref";

foreach my $i (sort keys %sample)

{

print "\tgenetype($i)\tref_depth/alt_depth($i)\tmutation_frequence($i)";

}

print "\n";

my %alt2;

foreach my $i (sort keys %alt)

{

my @a=split /\t/,$i;

foreach my $k (sort keys %{$alt{$i}})

{

$alt2{$a[0]}{$a[1]}{$k}=1;

}

}

my @bed;

my (%hash,%index);

foreach my $i (sort keys %alt2)

{

$index{$i}=0;

foreach my $k (sort {$a<=>$b} keys %{$alt2{$i}})

{

foreach my $l (sort {$a<=>$b} keys %{$alt2{$i}{$k}})

{

push @{$hash{$i}},[$k,$l];

push @bed,"$i\t$k\t$l";

}

}

}

my %alt3;

foreach my $bl(@bed)

{

my @a=split /\t/,$bl;

next unless exists $hash{$a[0]};

for(my $i=$index{$a[0]};$i<@{$hash{$a[0]}};$i++)

{

if($a[2]<$hash{$a[0]}[$i][0]){last;}

if ($a[1] > $hash{$a[0]}[$i][1]){$index{$a[0]}=$i;next};

unless($a[1] eq $hash{$a[0]}[$i][0] && $a[2] eq $hash{$a[0]}[$i][1])

{

$alt3{$bl}=1;

}

=cut

my $flag_tmp=0;

foreach my $k (sort keys %{$alt_type{"$a[0]\t$hash{$a[0]}[$i][0]"}{$hash{$a[0]}[$i][1]}})

{

if ($k =~/ins/)

{

$k=~s/ins//g;

my $len=length $k;

if ( $a[1] >= $hash{$a[0]}[$i][0] && $a[1] <= $hash{$a[0]}[$i][0]+$len)

{

$alt3{$bl}=1;

}

}

elsif ($q

}

=cut

}

}

foreach my $i (sort keys %alt)

{

foreach my $k (sort keys %{$alt{$i}})

{

next if (exists $alt3{"$i\t$k"});

print "$i\t$k\t$ref{$i}{$k}";

foreach my $j (sort keys %sample)

{

my $genetype;

if (exists $alt{$i}{$k}{$j})

{

$genetype="$alt{$i}{$k}{$j}";

}

elsif (exists $depth{$i}{$j})

{

if ($depth{$i}{$j} >=$depth_cut)

{

if ($flag{$i}{$k} eq "snp")

{

$genetype="$ref{$i}{$k}$ref{$i}{$k}\t$depth{$i}{$j}/0\t0";

}

else

{

$genetype="$ref{$i}{$k}\t$depth{$i}{$j}/0\t0";

}

}

else

{

$genetype="NA\tNA\tNA";

}

}

else

{

$genetype="NA\tNA\tNA";

}

print "\t$genetype";

}

print "\n";

}

}
