# Supplementary material for: Identification of Rice Blast Loss-of-Function Mutant Alleles in the Wheat Genome as a New Strategy for Wheat Blast Resistance Breeding
Source: Front Genet. 2021 May 19;12:623419. doi: 10.3389/fgene.2021.623419 (PMC8170139; doi:10.3389/fgene.2021.623419)
Supplement: Supplementary file 9 [file Data_Sheet_4.docx]

Table S1 Primer sequences for target gene amplification using TILLING by sequencing

| **Gene** | **Primer_F** | **Location** | **Primer_R** | **Location** |
| --- | --- | --- | --- | --- |
| TraesCS1A02G207700 | TTTGAGCATGAAGTGGCCAATATTT | -51~-27 | GAACTCTTATCCACACATCACGTTC | 6875~6899 |
| TraesCS1B02G221400 | ACCCAATGTAACCCAGAAATCCTTA | -640~-616 | AGCGTCAATTGTTACCTTCAAATGT | 3850~3874 |
| TraesCS1D02G211000 | ATACCTGAGGTGACATAAGTTCACC | -242~-218 | GTTAAGGTTGGCAAAGTATAGCTCC | 4618~4642 |
| TraesCS7A01G160700 | TCTCATCCGATTTAGGTCGAGTAAC | -219~-195 | CTGTTTGCTGAATCGTACACTGTAT | 1201~1225 |
| TraesCS7B01G065700 | TTATCTTCTGGGGTAGTGACTTTGG | -89~-65 | TTCACTTCTTCTCATGTCCTCATCA | 1356~1380 |
| TraesCS7D01G161800 | CTAATTGCCTACTAATTCAGGCTGC | -436~-412 | CTTCAAGCTCCTCATTGCTGAATAG | 967~991 |

Table S2 Sequence identity of rice blast S genes orthologs in wheat

| **Rice blast S gene** | **Gene ID** | **Conservative domain** | **Ortholog in wheat** | **Identities (Query length)** | **Percentage %** | **Expect** |
| --- | --- | --- | --- | --- | --- | --- |
| *Bsr-k1* | Os10g0548200 | TPR repeat | TraesCS1A02G207700 | 1187/1551 (9357) | 77 | 0 |
|  |  | TPR repeat | TraesCS1B02G221400 | 1173/1544 (9357) | 76 | 0 |
|  |  | TPR repeat | TraesCS1D02G211000 | 1180/1546 (9357) | 76 | 0 |
| *Bsr-d1* | Os03g32230 | C2H2-type zinc finger | TraesCS7A01G160700 | 459/602 (1097) | 76 | 7E-114 |
|  |  | C2H2-type zinc finger | TraesCS7B01G065700 | 453/602 (1097) | 75 | 4E-111 |
|  |  | C2H2-type zinc finger | TraesCS7D01G161800 | 454/607 (1097) | 75 | 2E-108 |

Table S3 SNPs identified in intronic regions of Bsr-k1 wheat orthologs

| **Line** | **Region** | **Allele** |
| --- | --- | --- |
| TraesCS1A02G207700 |  |  |
| E624 | intron2 | G779A |
| E417 | intron2 | G799A |
| E1388 | intron2 | G1036A |
| E1194 | intron2 | C1061T |
| A194 | intron2 | G1116A |
| E752 | intron2 | G1132A |
| A89 | intron2 | G1197A |
| A53 | intron2 | C1330T |
| E367 | intron2 | G1407A |
| A56 | intron2 | C1435T |
| E054-9 | intron2 | C1510T |
| A81 | intron2 | G1693A |
| A63 | intron2 | C1964T |
| E1194 | intron2 | C2020T |
| E1286 | intron2 | G2115A |
| A293 | intron2 | C2166T |
| E313 | intron2 | G2204A |
| E1369 | intron2 | C2391T |
| E040-11 | intron2 | T2817A |
| E1115 | intron2 | G2938A |
| E1333 | intron2 | G3065A |
| E040-15 | intron2 | G3549A |
| A380 | intron2 | C3573T |
| E748 | intron2 | C3611T |
| E393 | intron2 | G3712A |
| E044-5 | intron2 | C3726T |
| E746 | intron2 | G3797A |
| E968 | intron2 | G3939A |
| E1355 | intron2 | C4080T |
| E046-9 | intron2 | G4135A |
| E980 | intron2 | C4143T |
| E1368 | intron2 | G4237A |
| E040-16 | intron2 | G4497A |
| E318 | intron2 | C4561T |
| E757 | intron2 | G4626A |
| A353 | intron2 | C4697T |
| E978 | intron3 | G4942A |
| E504 | intron3 | C4985T |
| E054-4 | intron5 | G5342A |
| E1356 | intron5 | C5361T |
| TraesCS1B02G221400 |  |  |
| A403 | intron2 | G590A |
| E270 | intron2 | G830A |
| E1166 | intron2 | C831T |
| E630 | intron2 | G914A |
| E97 | intron2 | C933T |
| E046-7 | intron2 | G1041A |
| E69 | intron2 | G1362A |
| A165 | intron2 | G1491A |
| E1023 | intron2 | C1518T |
| E909 | intron2 | G1631A |
| E1362 | intron2 | G1636C |
| E398 | intron2 | G1636C |
| E367 | intron2 | C1867T |
| E045-5 | intron2 | G2002A |
| E045-4 | intron2 | C2041T |
| A146 | intron2 | C2050T |
| E140 | intron2 | G2108A |
| E259 | intron2 | C2116T |
| E48 | intron2 | C2170T |
| E478 | intron2 | C2240T |
| E1418 | intron2 | G2328A |
| E1266 | intron2 | G2492A |
| E954 | intron4 | C2825T |
| E751 | intron5 | G2994A |
| E047-4 | intron5 | C3011T |
| E047-3 | intron5 | C3011T |
| E049-6 | intron5 | C3011T |
| E131 | intron6 | G3227A |
| TraesCS1D02G211000 |  |  |
| E040-16 | intron1 | G479A |
| E1304 | intron2 | G846A |
| E904 | intron2 | G1040A |
| E924 | intron2 | G1061A |
| E72 | intron2 | C1078T |
| E043-3 | intron2 | T1148C |
| E039-9 | intron2 | T1148C |
| E021-8 | intron2 | G1163T |
| E615 | intron2 | C1360T |
| E880 | intron2 | T1449C |
| E912 | intron2 | T1449C |
| E1375 | intron2 | C1479T |
| E572 | intron2 | C1544T |
| A139 | intron2 | G1568A |
| E1411 | intron2 | G1650A |
| E828 | intron2 | G1957A |
| E1187 | intron2 | G1968A |
| E1279 | intron2 | C2147T |
| E216 | intron2 | G2183A |
| A201 | intron2 | G2230A |
| E1121 | intron2 | T2330C |
| E638 | intron2 | T2330C |
| E634 | intron2 | T2330C |
| E864 | intron2 | T2330C |
| E860 | intron2 | T2330C |
| E619 | intron2 | G2380A |
| E1332 | intron2 | G2532A |
| E1396 | intron2 | C2630T |
| E029-7 | intron2 | A2631G |
| E054-13 | intron2 | A2631G |
| E023-11 | intron2 | A2631G |
| E029-6 | intron2 | A2631G |
| E1284 | intron5 | C3173T |
| E782 | intron5 | C3197T |
| A55 | intron5 | C3197T |
| E054-14 | intron6 | G3396A |

Table S4 The sequence of specific primers for mutant validation in M3 generation

| **Gene** | **Line** | **Primer_F** | **Primer_R** |
| --- | --- | --- | --- |
| TraesCS1A02G207700 | E758 | TCTATGCTTTAACAATCGCA | AAAGGCCCCAGTAGTTACG |
|  | E325 | AGCTTTGCACCTCACTCCC | GGTTACCTAGATTGGTGATC |
| TraesCS1B02G221400 | E038-16 | GCGGTGGTGTTTTGACGCG | TGGCTTTGCTTCCATAGACTCT |
|  | E786 | GTTGATGAAACCCAAGTCATG | CACAGGAAAGGTTTCCAGT |
|  | E1294 | GTTGATGAAACCCAAGTCATG | CACAGGAAAGGTTTCCAGT |
|  | E410 | GTTGATGAAACCCAAGTCATG | CACAGGAAAGGTTTCCAGT |
| TraesCS1D02G211000 | E91 | GGTGGTGTTTTTGATGCG | GTCGCTGATTTATAGAAACA |
|  | E60 | AAAAAATTTGCGCTGCTGCG | CGATCAAAGCAAAGACCCG |
|  | E315 | AAAAAATTTGCGCTGCTGCG | CGATCAAAGCAAAGACCCG |
|  | E724 | TCCCGGGTCTTTGCTTTGAT | AGCTTCCCAAAGGAGTGAGGT |
| TraesCS7A02G160700 | E038-14 | CTTCTCATGTCCTCATCACCC | GACAGTCAACAATCCACCCAGCTC |
|  | E038-6 | CTGCTACCCACCTGCGTGATCGA | GCCGGGCGCCACACTCTCGCT |
| TraesCS7D02G161800 | A42 | ACTGCTGATCACGTAGGCA | GTAAAAGATGTATGCTCCA |
|  | A196 | ACTGCTGATCACGTAGGCA | GTAAAAGATGTATGCTCCA |
|  | A259 | ACTGCTGATCACGTAGGCA | GTAAAAGATGTATGCTCCA |
|  | E024-3 | ACTGCTGATCACGTAGGCA | GTCGGGAAGTACCGTAGCTC |
|  | E044-3 | ACCAAGTCCAAAGTTCCAAACC | GCAGCCGCGCAGCTGAAT |

Table S5 The number of amino acid changes and their respective proportion in each of the secondary structure forms of BRS-K1 wheat ortholog mutants

| **Gene** | **TraesCS1A02G207700** | | |  | **TraesCS1B02G221400** | | | | |  | **TraesCS1D02G211000** | | | | |
| --- | --- | --- | --- | --- | --- | --- | --- | --- | --- | --- | --- | --- | --- | --- | --- |
| **Line** | **WT** | **E758** | **E325** |  | **WT** | **E038-16** | **E786** | **E1294** | **E410** |  | **WT** | **E91** | **E60** | **E315** | **E724** |
| Alpha helix | 653, 56.59% | 652, 56.50% | 652, 56.50% |  | 651, 56.46% | 652, 56.55% | 655, 56.81% | 653, 56.63% | 650, 56.37% |  | 638, 55.43% | 638, 55.43% | 58, 53.70% | 637, 55.34% | 162, 65.32% |
| 310 helix | 0, 0.00% | 0, 0.00% | 0, 0.00% |  | 0, 0.00% | 0, 0.00% | 0, 0.00% | 0, 0.00% | 0, 0.00% |  | 0, 0.00% | 0, 0.00% | 0, 0.00% | 0, 0.00% | 0, 0.00% |
| Pi helix | 0, 0.00% | 0, 0.00% | 0, 0.00% |  | 0, 0.00% | 0, 0.00% | 0, 0.00% | 0, 0.00% | 0, 0.00% |  | 0, 0.00% | 0, 0.00% | 0, 0.00% | 0, 0.00% | 0, 0.00% |
| Beta bridge | 0, 0.00% | 0, 0.00% | 0, 0.00% |  | 0, 0.00% | 0, 0.00% | 0, 0.00% | 0, 0.00% | 0, 0.00% |  | 0, 0.00% | 0, 0.00% | 0, 0.00% | 0, 0.00% | 0, 0.00% |
| Extended strand | 68, 5.89% | 68, 5.89% | 68, 5.89% |  | 73, 6.33% | 74, 6.42% | 70, 6.07% | 73, 6.33% | 73, 6.33% |  | 80, 6.95% | 80, 6.95% | 2, 1.85% | 81, 7.04% | 7, 2.82% |
| Beta turn | 0, 0.00% | 0, 0.00% | 0, 0.00% |  | 0, 0.00% | 0, 0.00% | 0, 0.00% | 0, 0.00% | 0, 0.00% |  | 0, 0.00% | 0, 0.00% | 0, 0.00% | 0, 0.00% | 0, 0.00% |
| Bend region | 0, 0.00% | 0, 0.00% | 0, 0.00% |  | 0, 0.00% | 0, 0.00% | 0, 0.00% | 0, 0.00% | 0, 0.00% |  | 0, 0.00% | 0, 0.00% | 0, 0.00% | 0, 0.00% | 0, 0.00% |
| Random coil | 433, 37.52% | 434, 37.61% | 434, 37.61% |  | 429, 37.21% | 427, 37.03% | 428, 37.12% | 427, 37.03% | 430, 37.29% |  | 433, 37.62% | 433, 37.62% | 48, 44.44% | 433, 37.62% | 79, 31.85% |
| Ambiguous states | 0, 0.00% | 0, 0.00% | 0, 0.00% |  | 0, 0.00% | 0, 0.00% | 0, 0.00% | 0, 0.00% | 0, 0.00% |  | 0, 0.00% | 0, 0.00% | 0, 0.00% | 0, 0.00% | 0, 0.00% |
| Other states | 0, 0.00% | 0, 0.00% | 0, 0.00% |  | 0, 0.00% | 0, 0.00% | 0, 0.00% | 0, 0.00% | 0, 0.00% |  | 0, 0.00% | 0, 0.00% | 0, 0.00% | 0, 0.00% | 0, 0.00% |

File 1 The VCF file of six target fragments in the pooled samples

File 2 The Perl scripts written in the study to call SNPs from the VCF file.

File 3 An example of mutant discovery of gene TracesCS1A02G207700 in pooled plate
